# Loss of Function of Mutant IDS Due to Endoplasmic Reticulum-Associated Degradation: New Therapeutic Opportunities for Mucopolysaccharidosis Type II

**DOI:** 10.3390/ijms222212227

**Published:** 2021-11-12

**Authors:** Koji Matsuhisa, Kazunori Imaizumi

**Affiliations:** Department of Biochemistry, Institute of Biomedical & Health Sciences, Hiroshima University, Hiroshima 734-8553, Japan

**Keywords:** endoplasmic reticulum-associated degradation, iduronate-2-sulfatase, mucopolysaccharidosis type II, lysosomal storage disorder

## Abstract

Mucopolysaccharidosis type II (MPS II) results from the dysfunction of a lysosomal enzyme, iduronate-2-sulfatase (IDS). Dysfunction of IDS triggers the lysosomal accumulation of its substrates, glycosaminoglycans, leading to mental retardation and systemic symptoms including skeletal deformities and valvular heart disease. Most patients with severe types of MPS II die before the age of 20. The administration of recombinant IDS and transplantation of hematopoietic stem cells are performed as therapies for MPS II. However, these therapies either cannot improve functions of the central nervous system or cause severe side effects, respectively. To date, 729 pathogenetic variants in the *IDS* gene have been reported. Most of these potentially cause misfolding of the encoded IDS protein. The misfolded IDS mutants accumulate in the endoplasmic reticulum (ER), followed by degradation via ER-associated degradation (ERAD). Inhibition of the ERAD pathway or refolding of IDS mutants by a molecular chaperone enables recovery of the lysosomal localization and enzyme activity of IDS mutants. In this review, we explain the IDS structure and mechanism of activation, and current findings about the mechanism of degradation-dependent loss of function caused by pathogenetic IDS mutation. We also provide a potential therapeutic approach for MPS II based on this loss-of-function mechanism.

## 1. Background of Mucopolysaccharidosis Type II (MPS II)

Mucopolysaccharidoses (MPSs) are a group of lysosomal storage disorders caused by the deficiency of enzymes involved in the metabolism of glycosaminoglycans (GAGs) in the lysosome [1]. Seven types of MPS are categorized based on a lack of or defect in one of 11 specific lysosomal enzymes. MPS II, also known as Hunter syndrome, is the most frequent type of MPS, accounting for 53–58.1% of all MPS cases [2]. MPS II is closely related to MPS type I (MPS I), accounting for 14.8–16.2% of cases [2]. MPS II and MPS I are caused by the genetic absence or dysfunction of the lysosomal enzymes iduronate-2-sulfatase (IDS) and α-L-iduronidase (IDUA), respectively [3,4,5,6]. These enzymes sequentially degrade heparan sulfate and dermatan sulfate of GAGs. First, IDS catalyzes the hydrolysis of the sulfate groups in heparan sulfate and dermatan sulfate. Then, IDUA removes a single α-L-iduronyl residue from these substrates. Dysfunction of IDS or IDUA results in the accumulation of GAGs in the lysosomes of cells and tissues throughout the body. This accumulation of GAGs contributes to the pathology of MPS II and MPS I. MPS II patients and MPS I patients share similar symptoms because of the accumulation of the same substrates. However, there are some differences between the pathologies of these MPSs [7].

MPS II is an X-linked genetic disorder, with an estimated incidence of 0.3–0.7 per 100,000 births [8]. Patients with MPS II exhibit mental retardation and systemic manifestations including coarse facial features, hearing difficulty, skeletal deformities, rigid joints, a thick skin, hepatosplenomegaly, and valvular heart disease. MPS I patients present similar symptoms, but epidermal symptoms are infrequently observed [7]. MPS II is classified into two types based on the clinical phenotypes. The “severe” early-onset form, which accounts for approximately 60% of patients with MPS II, exhibits cognitive impairment, behavioral difficulties, and frequent epileptic seizures [9]. Behavioral difficulties and seizures are frequently observed in MPS II patients with central nervous system (CNS) involvement, but they are rare in patients with MPS I [7]. The majority of patients die from obstructive airway disease or cardiac failure before the age of 20 [6,10,11]. The second type of MPS II, the “attenuated” late-onset form, presents normal or slightly impaired cognitive function, and the patients normally survive into late adulthood [3].

To date, 729 pathogenetic variants in the *IDS* gene have been reported {Human Gene Mutation Database (HGMD) Professional 2021.2, http://www.hgmd.cf.ac.uk, accessed on 28 October 2021. The pathogenetic variants of the *IDS* gene include missense/nonsense variants, deletions, frameshift variants, and splicing abnormalities. No definitive genotype–phenotype correlation has been proposed because of the rarity of the disease and the very high number of different variants reported in the *IDS* gene [12]. All pathogenetic variants of the *IDS* gene in patients with MPS II are loss-of-function variants, leading to the pathogenesis of the disease. In addition to mutations at active sites, mutations at other sites also cause loss of function of IDS [13,14]. Overall, 219 (89%) out of 247 pathogenetic missense mutations of IDS were not located at the active sites (HGMD Professional 2021.2).

## 2. Current Therapy for MPS II and Its Difficulties

Enzyme replacement therapy (ERT) and hematopoietic stem cell (HSC) transplantation have been performed on patients with MPS II [12]. The concepts of these therapies have been demonstrated via experiments using fibroblasts from patients affected by MPS I or MPS II [15]. IDS secreted from cells of patients with MPS I were incorporated into IDS-deficient cells of patients with MPS II via the mannose-6-phosphate receptor [15,16]. The incorporated IDS translocated into the lysosomes, where it catalyzed the degradation of GAGs. This phenomenon is called “cross-correction.”

At present, two recombinant IDSs, Idursulfase and Idursulfase beta, are available in ERT for MPS II [17,18]. ERT achieves somatic improvements including in urine GAG level, liver size, hearing, and joint range of motion [19,20]. However, no improvements in respiratory function, the eyes, skeleton, or CNS were found in patients with severe MPS II [20]. The limited efficacy of ERT in some tissues can be explained by the low bioavailability of the enzyme because of the low vascularization of tissues such as bone, cartilage, and cardiac valves, and by the presence of biological barriers such as the blood–brain barrier (BBB), inhibiting CNS treatment [21].

In HSC transplantation, healthy donor stem cells are obtained from bone marrow, cord blood, or peripheral blood stem cells, and are transplanted into patients to provide cross-correction of the enzyme in deficient tissues [22,23,24]. Because HSCs can permeate the BBB, HSCs derived from donor cells would be able to secrete the active enzymes that would be captured by receptor neurons [25]. A clinical study showed that speech deterioration was significantly less severe in patients who underwent HSC transplantation than in an untreated group [26]. Moreover, when full donor chimerism was achieved, a single intervention could provide a durable lifelong enzyme source to the affected patient [27]. Nevertheless, graft-versus-host disease occurred in eight (9%) out of 85 cases, and nine (8%) patients died from transplantation-associated complications [28]. Although improvements in donor selection and regimens have decreased the mortality rate of HSC transplantation for MPSs to less than 5% [29], this therapy in MPS II is not currently recommended as a treatment option. Other approaches are also needed for the safe and effective treatment of MPS II.

## 3. Biochemical Characteristics of IDS

**Figure 1:**
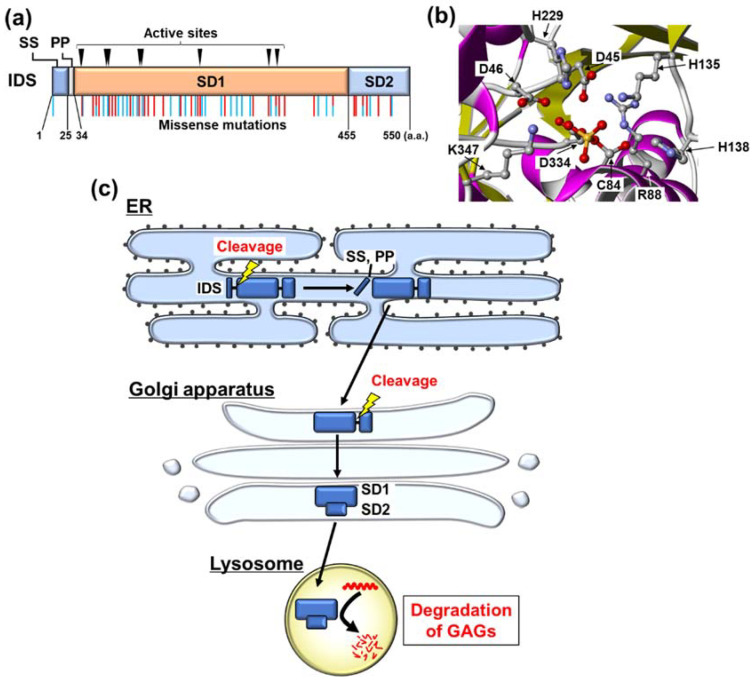
The peptide features of iduronate-2-sulfatase (IDS) and its processing steps in organelles. (**a**) Domain structure, active sites, and representative pathogenetic mutations of IDS. IDS protein consists of a signal peptide (SS), a propeptide (PP), subdomain 1 (SD1), and subdomain 2 (SD2). SD1 contains nine active-site residues: D45, D46, C84, R88, K135, H138, H229, D334, and K347. Representative residues that are mutated are shown as sticks on the structures of IDS. Red: severe type, Blue: attenuated type, Red and Blue: both attenuated and severe types are reported. (**b**) Crystal structure of active site of IDS protein. Only side chains of active-site residues are presented with balls and sticks. Cartoon diagram colored by secondary structure. Image from the Protein Data Bank Japan (https://pdbj.org/, accessed on 1 October 2021) of PDB ID 5FQL [13], created with Molmil Viewer (© Protein Data Bank Japan (PDBj) licensed under CC-BY-4.0 International). (**c**) Processing of IDS. In the ER, SS and PP are cleaved from IDS. The C-terminal fragments are translocated into the Golgi apparatus, followed by undergoing proteolytic cleavage between SD1 and SD2. Cleaved SD1 and SD2 form a complex via non-covalent bonds. The complex translocates into the lysosome, where it catalyzes the degradation of glycosaminoglycans (GAGs).

IDS is a protein of 550 amino acids in length that displays high homology with the sulfatase protein family [30,31]. It localizes in the lysosome. The enzyme consists of an N-terminal signal peptide, a propeptide, and two subdomains, subdomain 1 (SD1) and subdomain 2 (SD2) (Figure 1a) [13]. SD1 contains nine active-site residues (Figure 1a,b) [13]. IDS contains two intramolecular disulfide bonds: one between Cys171 and Cys184, the other between Cys422 and Cys432 [13]. Maturation of IDS involves post-translational modifications including proteolytic cleavage, glycosylation, and the conversion of the Cys84 residue [14]. IDS contains eight N-glycosylated residues, with the N-glycosylation at Asn208 being important for its lysosomal targeting [32]. The Cys84 residue of IDS is modified to formylglycine [13]. This modification is required for the catalytic activity of IDS [33]. Following the synthesis of IDS, the protein undergoes shedding of the signal peptide and propeptide in the endoplasmic reticulum (ER) (Figure 1c). The Cys84 residue of IDS is oxidized to formylglycine by the ER-resident formylglycine-generating enzyme. Additionally, IDS undergoes N-glycosylation at eight Asn residues in the ER. Subsequently, the C-terminal fragment containing SD1 and SD2 translocates to the Golgi apparatus. This fragment is further cleaved between SD1 and SD2, which are modified by complex types of carbohydrates at the Golgi apparatus [34]. SD1 and SD2 form a complex via non-covalent bonds [13]. This complex moves into the lysosome, where it catalyzes the degradation of GAGs as a mature form of IDS. 

The variant spectrum of the *IDS* gene is diverse and ranges from small point variants, deletions, and insertions to truncations, large deletions, and complex rearrangements [13]. A large proportion of pathogenetic mutations associated with MPS II are missense mutations randomly distributed throughout the protein sequence including the active sites, N-glycosylation sites, and around both of these residues (Figure 1a). The missense mutations are likely to result in protein misfolding, catalytic inactivation, premature degradation, or failure of lysosomal targeting. These abnormalities lead to loss of function of the pathogenetic IDS mutants. Several expression studies have shown that a small amount of the mature form is produced from the IDS variants found in patients with attenuated-type MPS II [14,35]. The attenuated-type mutants have residual enzyme activity. In contrast, the mature forms are not observed in the cells expressing the severe-type mutant IDS, resulting in almost completely deficient enzyme activity [14,35].

## 4. Misfolding of IDS Caused by Its Mutation

Various studies have suggested that pathogenetic IDS mutants are misfolded in the ER [13,36,37]. A previous study on an X-ray crystal structure of IDS revealed that several mutations, namely, L41P, L67P, L72P, L73F, L221P, L314P/H, L403R, and L410P, would introduce main-chain geometry distortion or buried steric clashes, disrupting the local secondary structure in the core of SD1 [13]. This structural change indicates that mutant IDS exhibits a misfolded structure. Meanwhile, biochemical and biological studies have also suggested the possibility of the unfolding of IDS mutants. Proteomic analysis of a mouse brain showed that IDS interacts with the ER-resident chaperone heat shock protein 90 [36]. Chaperone proteins bind tightly to unfolded proteins. Therefore, binding of the chaperone protein to IDS suggests that IDS is prone to forming an unfolded structure. Another group reported that the spliced form of X-box binding protein 1 (XBP1), frequently used as a marker of ER stress, was upregulated in neuronal progenitor cells derived from induced pluripotent stem cells of patients with MPS II [37]. Induction of this ER stress marker is triggered by the accumulation of unfolded proteins in the ER [38], indicating that mutant IDS accumulates in the ER as an unfolded protein. Collectively, these studies suggest the possibility that mutations induce the misfolding of IDS proteins, leading to their loss of function.

## 5. Degradation of Mutant IDS by the ER-Associated Degradation (ERAD) System

Various pathological conditions including the expression of mutant proteins induce the accumulation of unfolded or misfolded proteins in the ER lumen. The proteins accumulated in the ER are degraded by the ubiquitin–proteasome system via a process known as ER-associated degradation (ERAD). In the ERAD pathway, misfolded proteins in the ER lumen are recognized by molecular chaperones and by chaperone-like lectins [39]. Subsequently, the misfolded proteins are exported into the cytosol via retrotranslocation. During retrotranslocation, the misfolded proteins are conjugated with a polyubiquitin chain by ubiquitin E3 ligases including hydroxymethyl glutaryl-coenzyme A reductase degradation protein 1 (HRD1). The modified proteins are removed from the ER and degraded by the cytoplasmic 26S proteasome.

**Figure 2:**
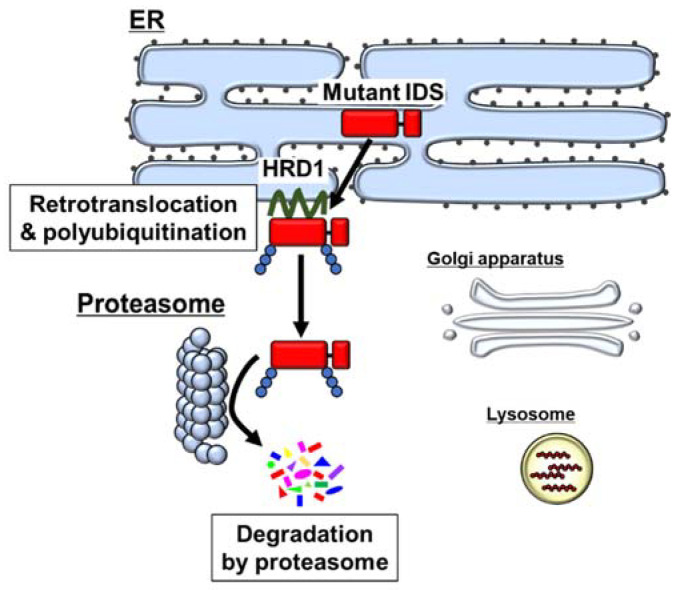
Degradation of IDS in the ERAD pathway. Mutant IDS accumulates in the ER, followed by retrotranslocation to the cytoplasm and polyubiquitination. The polyubiquitinated mutants are degraded by proteasomes.

Recent reports have revealed that IDS mutants accumulate in the ER, and are degraded by the ERAD pathway (Figure 2) [40,41]. Two types of IDS mutants, A85T (attenuated type) and R468Q (severe type), accumulate in the ER. Their lysosomal localization is rarely observed. The accumulated IDS mutants are polyubiquitinated by the ERAD-related ubiquitin E3 ligase HRD1 [40]. Subsequently, the mutants are degraded by proteasome. Inhibition of this degradation enables recovery of the lysosomal localization and enzyme activity of the A85T mutant with an approximately 1.3-fold increase [40]. It has been reported that in most lysosomal storage disorders, minimal increases of enzyme activity are sufficient to positively impact the phenotype [42]. Various studies on MPS II patients with attenuated or severe forms indicated that only a small improvement in enzyme activity drastically relieves the symptoms of MPS II [3,35]. Therefore, rescue of IDS mutants by the inhibition of ERAD may be sufficient to attenuate the pathology in patients with MPS II. One of the severe-type truncated mutants, W337X, was also reported to be degraded by the ERAD pathway, although the lysosomal localization and enzyme activity of the mutant under the inhibition of ERAD have not been determined [43]. Although further investigations are needed, other MPS II-related IDS mutants may also cause loss of function through degradation via the ERAD pathway. Inevitably, escape from degradation cannot rescue IDS mutants that completely lose their enzyme activity by mutation at the active sites. 

**Figure 3:**
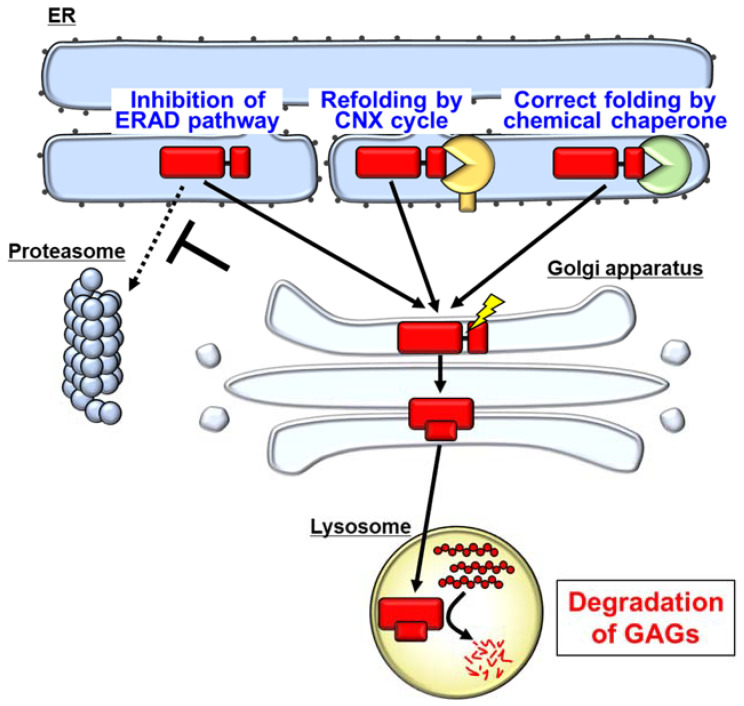
Potential approaches targeting IDS mutants accumulated in the ER for treating MPS II. Inhibition of ERAD-dependent degradation of IDS mutants increases the mutants that translocate to the Golgi apparatus and lysosomes. Correct folding by the calnexin (CNX) cycle or chemical chaperones allows IDS mutants to pass the ER quality control, the mutants subsequently moving into the lysosomes. They degrade accumulated GAGs, thereby attenuating the pathology in MPS II.

Calnexin (CNX) is one of the ER-resident chaperones. Unfolded proteins are retained in the ER, where they undergo advanced folding by binding repeatedly with CNX (CNX cycle) [44,45,46]. Kifunensine, an inhibitor of α-mannosidase I, promotes the binding of CNX and IDS mutants, and subsequently rescues the enzyme activity of A85T IDS [41]. This suggests that the refolding of IDS mutants in the CNX cycle recovers the enzyme activity (Figure 3). 

However, ERAD is one of the systems protecting against the accumulation of unfolded or misfolded proteins in the ER lumen. This abnormal accumulation of unfolded proteins is termed ER stress [47,48]. ER stress is involved in the onset of various diseases including neurodegenerative disorders and diabetes [49,50]. Long-term inhibition of ERAD may enhance ER stress, leading to the pathogenesis of ER stress-related diseases. Thus, ERAD inhibitors are currently not suitable as therapeutic agents to treat chronic diseases. Specific inhibitors of the ERAD-dependent degradation of pathogenetic IDS variants may have a therapeutic effect on MPS II without the side effects associated with ER stress.

Although ERAD-dependent degradation of pathogenetic IDS mutants seems to contribute to the mutants’ loss of function, few reports have addressed this loss of function. More research on various pathogenetic IDS variants and in vivo studies are needed to further understand the loss of function of IDS by its degradation via ERAD in MPS II.

## 6. Pharmacological Chaperones as a Possible Therapeutic Strategy for MPS II

Pharmacological chaperones correct the folding of misfolded proteins. Pharmacological chaperones have been used successfully in other lysosomal diseases because they provide many advantages such as being orally administrated and crossing the BBB [42]. Moreover, pharmacological chaperones are already in development for treating other MPSs [51]. Therefore, the refolding of pathogenetic IDS mutants by pharmacological chaperones may be a promising therapeutic strategy for MPS II (Figure 3). Indeed, it has been reported that one of the chemical chaperones, Δ-unsaturated 2-sulfouronic acid-N-sulfoglucosamine (D2S0), improves IDS activity and GAG accumulation in fibroblasts derived from MPS II patients [52]. D2S0 increased the enzyme activity of several types of mutated IDS, namely, N63D, L67P, A85T, R88H, Y108S, P231L, and L314P when these mutants were exogenously expressed in HEK293T cells [52]. Although the intracellular localization of the mutants in cells treated with D2S0 has not been analyzed, the reduction in GAG accumulation suggests that D2S0 may promote the translocation of IDS mutants into the lysosome. These results suggest that the refolding of mutant IDS by a pharmacological chaperone rescues the enzyme activity.

Pharmacological chaperone therapy and potential strategies targeting the refolding or degradation of IDS are based on the residual activity of mutant IDS. Therefore, these strategies cannot rescue IDS mutants that completely lack the enzyme activity. Indeed, both refolding by CNX and inhibition of ERAD did not rescue the enzyme activity of R468Q IDS [40,41]. The mutation in R468Q IDS causes severe misfolding, which leads to the complete loss of IDS function [13]. Although refolding or inhibition of the degradation is not effective for IDS mutants with no enzyme activity, these strategies will provide novel therapeutic options for patients with attenuated-type MPS II.

## 7. Rare Diseases and ERAD

To date, more than 6000 rare diseases have been reported worldwide, approximately 80% of which are caused by pathogenetic variants [53]. Missense variants are included among the variants in approximately 80% of the rare disease-associated genes [54]. Missense variants possibly cause misfolding or unfolding of the encoded proteins, which are then degraded by the ERAD pathway. Indeed, many studies using cell lines and mouse models have revealed that mutant proteins involved in many rare diseases including other lysosomal storage disorders are degraded by ERAD (Table 1) [55,56,57,58,59,60,61,62,63,64,65,66,67,68,69,70,71,72,73]. Several studies have suggested the possibility that, in some rare diseases, ERAD inhibition also rescues the function of the mutant proteins. For example, the effect of ERAD inhibition has been reported in a study on the lysosomal storage disorder Gaucher disease [55]. Knockdown of the ERAD component ERdj3 enabled recovery of the enzyme activity of L444P mutant glucocerebrosidase, an enzyme deficient in Gaucher disease. This indicates that degradation of the mutant glucocerebrosidase via the ERAD pathway is involved in the loss of function of the enzyme. Pharmacological chaperones rescue the function of several pathogenetic variants causing Gaucher disease and another lysosomal storage disorder, Fabry disease, in pre-clinical and clinical trials [57,58,59,60]. Notably, a pharmacological chaperone has already been approved for the treatment of Fabry disease [60]. This background suggests that correct folding by pharmacological chaperones rescues the functions of the pathogenetic mutants in these diseases. The effect of pharmacological chaperones also implies that loss of function of the mutants in lysosomal storage disorders is caused by degradation via the ERAD pathway. Similarly, it is considered that unfolded mutant proteins often lose their function by degradation via ERAD, even though the mutants retain residual function. Transmembrane proteins, secretory proteins, and proteins that localize inside organelles are transported via the ER. Therefore, in addition to the rare diseases listed in Table 1, loss of function of mutant proteins, except for the enzymes with mutations at active sites, may be caused by ERAD-dependent degradation in other rare diseases. This common mechanism underlying loss of function of mutant proteins may be a novel universal therapeutic target for most rare diseases.

## 8. Conclusions

Although pathogenetic IDS variants found in patients with attenuated-type MPS II normally display residual enzyme activity, these variants are degraded by the ERAD pathway. When the mutants translocate into the lysosome, mutants with residual activity have the potential to degrade GAGs in the lysosome. Refolding of the IDS mutants by the CNX cycle or pharmacological chaperones may be a novel strategy to allow the mutants to avoid degradation. Experimentally, inhibition of ERAD also rescues the activity of IDS mutants. However, modulation of the ERAD pathway may cause severe side effects because of its physiological significance. Therefore, inhibition of the ERAD pathway is unsuitable as a therapeutic strategy. Taking these findings together, quality control of IDS mutants in the ER may be a novel therapeutic target for ameliorating dysfunction of the mutants, potentially providing breakthroughs for treating MPS II.

## Figures and Tables

**Table 1:** ERAD-dependent degradation of responsible proteins in hereditary diseases.

Disease	ERAD Substrate	References
Gaucher disease	GCase	[55,56,57,58]
Fabry disease	α-Gal A	[59,60]
Tay-Sachs disease	HexA (α subunit)	[61]
Cystic fibrosis	CFTR	[62,63,64,65]
diabetes insipidus	AQP2	[66]
retinitis pigmentosa	rhodopsin	[67,68]
High blood pressure	vanin-1	[69]
Stargardt disease	ABCA4	[70]
Charcot-Marie-Tooth disease	PMP22	[71]
Type I Bartter syndrome	NKCC2	[72]
Type II Bartter syndrome	POMC	[73]

GCase: glucocerebrosidase, α-Gal A: α-galactosidase A, HexA: β-hexosaminidase A, CFTR: cystic fibrosis transmembrane conductance regulator, AQP2: aquaporin 2, ABCA4: ATP binding cassette subfamily A member 4, PMP22: peripheral myelin protein 22, NKCC2: sodium-potassium-chloride cotransporter 2, POMC: pro-opiomelanocortin.

## Abbreviations

ABCA4	ATP binding cassette subfamily A member 4
α-Gal A	α-galactosidase A
AQP2	aquaporin 2
BBB	Blood–brain barrier
CFTR	Cystic fibrosis transmembrane conductance regulator
CNS	Central nervous system
CNX	Calnexin
D2S0	Δ-unsaturated 2-sulfouronic acid-N-sulfoglucosamine
ER	Endoplasmic reticulum
ERAD	Endoplasmic reticulum-associated degradation
ERT	Enzyme replacement therapy
GAG	Glycosaminoglycan
GCase	Glucocerebrosidase
HexA	Β-hexosaminidase A
HGMD	Human Gene Mutation Database
HRD1	Hydroxymethyl glutaryl-coenzyme A reductase degradation protein 1
HSC	Hematopoietic stem cell
IDS	Iduronate-2-sulfatase
IDUA	α-L-iduronidase
MPS	Mucopolysaccharidosis
MPS I	Mucopolysaccharidosis type I
MPS II	Mucopolysaccharidosis type II
NKCC2	Sodium-potassium-chloride cotransporter 2
PMP22	Peripheral myelin protein 22
POMC	Pro-opiomelanocortin
PP	Propeptide
SD1	Subdomain 1
SD2	Subdomain 2
SS	Signal peptide
XBP1	X-box binding protein 1
